# Prostaglandin D_2_ Added during the Differentiation of 3T3-L1 Cells Suppresses Adipogenesis via Dysfunction of D-Prostanoid Receptor P1 and P2

**DOI:** 10.3390/life13020370

**Published:** 2023-01-29

**Authors:** Michael N. N. Nartey, Mitsuo Jisaka, Pinky Karim Syeda, Kohji Nishimura, Hidehisa Shimizu, Kazushige Yokota

**Affiliations:** 1 The United Graduate School of Agricultural Sciences, Tottori University, 4-101 Koyama-Minami, Tottori 680-8553, Japan; 2 Council for Scientific and Industrial Research-Animal Research Institute, Achimota, Accra P.O. Box AH20, Ghana; 3 Department of Life Science and Biotechnology, Shimane University, 1060 Nishikawatsu-Cho, Matsue 690-8504, Japan; 4 Institute of Agricultural and Life Sciences, Academic Assembly, Shimane University, 1060 Nishikawatsu-Cho, Matsue 690-8504, Japan; 5 Interdisciplinary Center for Science Research, Shimane University, 1060 Nishikawatsu-Cho, Matsue 690-8504, Japan

**Keywords:** PGD_2_, DP1, DP2, adipocyte, adipogenesis

## Abstract

We previously reported that the addition of prostaglandin, (PG)D_2_, and its chemically stable analog, 11-deoxy-11-methylene-PGD_2_ (11d-11m-PGD_2_), during the maturation phase of 3T3-L1 cells promotes adipogenesis. In the present study, we aimed to elucidate the effects of the addition of PGD_2_ or 11d-11m-PGD_2_ to 3T3-L1 cells during the differentiation phase on adipogenesis. We found that both PGD_2_ and 11d-11m-PGD_2_ suppressed adipogenesis through the downregulation of peroxisome proliferator-activated receptor gamma (PPARγ) expression. However, the latter suppressed adipogenesis more potently than PGD_2_, most likely because of its higher resistance to spontaneous transformation into PGJ_2_ derivatives. In addition, this anti-adipogenic effect was attenuated by the coexistence of an IP receptor agonist, suggesting that the effect depends on the intensity of the signaling from the IP receptor. The D-prostanoid receptors 1 (DP1) and 2 (DP2, also known as a chemoattractant receptor-homologous molecule expressed on Th2 cells) are receptors for PGD_2_. The inhibitory effects of PGD_2_ and 11d-11m-PGD_2_ on adipogenesis were slightly attenuated by a DP2 agonist. Furthermore, the addition of PGD_2_ and 11d-11m-PGD_2_ during the differentiation phase reduced the DP1 and DP2 expression during the maturation phase. Overall, these results indicated that the addition of PGD_2_ or 11d-11m-PGD_2_ during the differentiation phase suppresses adipogenesis via the dysfunction of DP1 and DP2. Therefore, unidentified receptor(s) for both molecules may be involved in the suppression of adipogenesis.

## 1. Introduction

Obesity caused by overnutrition used to be considered a problem specific to high-income countries, but it is now an increasing problem even in low- and middle-income countries, where the prevalence of obesity previously tended to be low [1]. Obesity is a problem because it causes the development of cardiovascular disease, type 2 diabetes, cancer, osteoarthritis, occupational disorders, and sleep apnea [1]. For instance, as early as 2001, it was predicted that because cardiovascular disease survival rates had become higher in industrialized countries than in non-industrialized countries, the number of patients with cardiovascular disease associated with obesity would increase, particularly in industrialized countries [2]. In addition, the current number of patients with type 2 diabetes associated with obesity is also predicted to increase, particularly in low- and middle-income countries. As a result, increases in the prevalence of nephropathy, atherosclerosis, neuropathy, and retinopathy due to type 2 diabetes are expected in these countries [2]. Thus, obesity, which is a major cause of health problems and a reduced quality of life, has gone from being a relatively minor public health problem primarily affecting affluent societies to a major threat to public health worldwide. To prevent obesity with excess adipose tissue, which has become such a major threat to public health, it is important to understand the mechanisms of adipogenesis. Immortal preadipocyte cell lines, such as 3T3-L1 cells, have been established with the goal of elucidating the mechanisms of adipogenesis [3,4]. The stages of adipogenesis comprise growth, differentiation, and maturation, and incubating confluent 3T3-L1 preadipocytes in medium containing 3-isobutyl-1-methylxanthine (IBMX), dexamethasone, and insulin (MDI) during differentiation can initiate adipogenesis.

Peroxisome proliferator-activated receptors (PPAR)s are ligand-activated transcription factors that belong to the nuclear hormone receptor superfamily and function as heterodimers with the retinoid X receptor (RXR). Three types of PPARs were isolated, PPARα, PPARδ (also known as PPARβ, NUC1, and FAAR), and PPARγ, each with different reported physiological functions [5,6]. PPARγ has two protein isoforms, PPARγ1 and PPARγ2, which result from alternative promoter usage and differential splicing at the 5′ end of the gene [7,8]. Multiple lines of evidence have been reported regarding the importance of PPARγ, one of the master regulators of adipogenesis, during the process of differentiation from preadipocytes to adipocytes, as described below. The findings of the initial in vitro studies showed that the ligand-dependent activation of ectopically expressed PPARγ2 accelerates adipogenesis in fibroblast cell lines [9]. Furthermore, the findings of the studies in which PPARγ antagonists and dominant-negative forms of the receptor were used showed that the loss of the receptor function is related to a decreased ability to differentiate adipocytes [10,11,12]. Although it was difficult to establish the importance of adipogenesis in vivo because the genetic disruption of PPARγ confers an embryonic lethal phenotype [13,14], the data obtained from chimeric animals generated using an improved transgenic technique confirmed the importance of PPARγ during adipogenesis in mice [13,14].

PPARγ can upregulate the expression of a variety of adipocyte-specific genes related to adipogenesis, including adiponectin and lipoprotein lipase (LPL) [15,16]. Adiponectin is known as a fat-derived hormone, and genetic mutations in the adiponectin gene that result in low plasma adiponectin levels have been reported to be associated with metabolic syndrome, including insulin resistant, diabetes, and atherosclerotic disease [17]. LPL is known as an enzyme that hydrolyzes triacylglycerol (TAG) in lipoprotein particles into fatty acids and monoglycerol [18]. In addition, LPL has been reported to be involved in the uptake mechanism of TAG in medium into 3T3-L1 cells, resulting in the accumulation of TAG in the cells [19]. Adiponectin and LPL have a functional PPAR-responsive element (PPRE) in their promoters, and the PPARγ/RXR heterodimer was shown to bind directly to these PPREs and regulate their expression [15,16]. Therefore, these two genes are frequently used as adipogenic marker genes, associated with PPARγ expression levels.

The biosynthesis of prostaglandins (PG)s occurs via multiple enzymatically controlled reactions. This process is initiated by the release of arachidonic acid synthesized from linoleic acid, one of the ingested n-6 polyunsaturated fatty acids (n-6 PUFA), from membrane phospholipids via the catalytic action of phospholipases. Subsequently, arachidonic acid is then converted to PGG_2_, and then PGH_2_, through the action of PG endoperoxide synthase, cyclooxygenase (COX). COX has two distinct isozymes, COX-1 and -2, which are differentially regulated [20]. The unstable PGH_2_ intermediates generated via COX-1 or -2 are substrates for certain PG synthases and catalyze the formation of bioactive PGs [21]. PGs are broadly classified into two categories: anti-adipogenic PGs and pro-adipogenic PGs. Anti-adipogenic PGs include PGE_2_ [22,23,24] and PGF_2α_ [25,26], which inhibit adipogenesis by activating EP4 and FP receptors, respectively [23,24,25,26]. Pro-adipogenic PGs include the PGJ_2_ derivatives, 15-deoxy-Δ^12,14^-PGJ_2_ and Δ^12^-PGJ_2_ [27,28,29,30], and PGI_2_ [31,32]. In addition to the IP receptor being a known receptor for PGI_2_ [32], the activation of the IP receptor is suggested to activate PPARγ in HEK293 cells [33].

PGD_2_ is biosynthesized via lipocalin-type PGD synthase (L-PGDS), which is preferentially expressed in adipocytes from PGH_2_ produced from arachidonic acid via COXs [21] and is also known as a precursor to PGJ_2_ derivatives [34]. In addition to the increased transcriptional activity of PPARγ induced by PGD_2_ [27,28], the addition of PGD_2_ during the maturation phase promotes MDI-induced adipogenesis by binding and activating D-prostanoid 1 (DP1) and/or 2 receptor (DP2, also known as a chemoattractant receptor-homologous molecule expressed on TH 2 cells) [35,36]. Based on these reports, PGD_2_ is considered to be pro-adipogenic. Nevertheless, L-PGDS is involved in PGD_2_ biosynthesis and is preferentially expressed in adipocytes. Antisense L-PGDS reduces the endogenous expression of L-PGDS and promotes adipogenesis when stably expressed in 3T3-L1 preadipocytes [37]. Conversely, stably overexpressed L-PGDS impairs adipogenesis in 3T3-L1 preadipocytes [38]. Furthermore, the altered L-PGDS expression in 3T3-L1 preadipocytes is reflected by the abundance of PGD_2_ and PGJ_2_ derivatives [37,38]. These findings regarding L-PGDS support the notion that incubating 3T3-L1 cells with PGD_2_ during the differentiation phase inhibits MDI-induced adipogenesis. Furthermore, PGD_2_ undergoes nonenzymatic dehydration and is readily converted to PGJ_2_ derivatives [34], whereas the chemically stable PGD_2_ analog, 11-deoxy-11-methylene-PGD_2_ (11d-11m-PGD_2_) with an exocyclic methylene, instead of being in an 11-keto group, is considered to resist spontaneous conversion to PGJ_2_ derivatives. In fact, we were able to generate an antibody against PGD_2_ using 11d-11m-PGD_2_ [34]. Based on the previous reports, we considered 11d-11m-PGD_2_ to facilitate the analysis of the mechanism through which PGD_2_ affects MDI-induced adipogenesis. Therefore, in the present study, we aimed to determine whether the incubation of 3T3-L1 cells with PGD_2_ or 11d-11m-PGD_2_ during the differentiation phase inhibits MDI-induced adipogenesis during the maturation phase. In addition, the crosstalk between PGD_2_ or 11d-11m-PGD_2_ and IP signaling that promotes the pro-adipogenic effect was also analyzed.

## 2. Materials and Methods

### 2.1. Materials

The following were obtained from the respective suppliers: Dulbecco’s modified Eagle medium containing 25 mM HEPES, penicillin G potassium salt, streptomycin sulfate, dexamethasone, fatty acid-free bovine serum albumin, and recombinant human insulin (Sigma-Aldrich Corp., St. Louis, MO, USA); L-ascorbic acid phosphate magnesium salt n-hydrate, 3-isobutyl-1-methylxanthine (IBMX), and Triglyceride E-Test Kits (Wako Pure Chemical Industries Ltd., Osaka, Japan); fetal bovine serum (FBS) (MP Biomedicals, Solon, OH, USA); PGD_2_, 11d-11m-PGD_2_, MRE-269, BW245C, and 15*R*-15-methyl-PGD_2_ (15*R*-15m-PGD_2_) (Cayman Chemical (Ann Arbor, MI, USA); M-MLV reverse transcriptase (Ribonuclease H minus, point mutant) and polymerase chain reaction (PCR) Master Mix (Promega Corp., Madison, WI, USA).

### 2.2. Cell Culture of 3T3-L1 Cells and Induction of Adipogenesis

Mouse 3T3-L1 pre-adipogenic cells (JCRB9014; JCRB Cell Bank, Osaka, Japan) at the growth phase were seeded at a density of 1 × 10^5^ or 2 × 10^5^ in 35 or 60 mm dishes containing 2 or 4 mL, respectively, in a growth medium (GM) comprising DMEM containing HEPES supplemented with 10% fetal bovine serum (FBS), penicillin G (100 units/mL), streptomycin sulfate (100 μg/L), and ascorbic acid (200 μM). Then, they were incubated at 37 °C under 7% CO_2_ until they reached confluence. Confluent monolayers were incubated with differentiation medium (DM) comprising GM supplemented with dexamethasone (1 μM), IBMX (0.5 mM), and insulin (10 μg/mL) for 48 h to induce differentiation into adipocytes. The cells were then cultured for 6 to 10 days in a maturation medium (MM; GM supplemented with 5 μg/mL of insulin). The medium was replaced with fresh MM, every 2 days for 10 days, to promote fat storage in the adipocytes during maturation. We examined the effects of various agents on adipogenesis during differentiation by incubating confluent cell monolayers in DM, supplemented with test compounds, for 48 h, followed by the standard maturation protocol. The test compounds were dissolved in ethanol and added to the DM to a final ethanol concentration of 0.2%.

### 2.3. Quantitation of Intracellular Triacylglycerols and Proteins

The cultured mature adipocytes were harvested, suspended in phosphate-buffered saline (PBS) without Ca^2+^ and Mg^2+^ (PBS [-]), supplemented with 0.05% trypsin and 0.53 mM EDTA, and incubated at 37 °C for 5 min. The cell suspensions were washed with PBS (-), divided into two portions, and homogenized in 25 mM Tris-HCl buffer (pH 7.4) containing 1 mM EDTA and 1 N NaOH. Intracellular triacylglycerol (TAG) accumulation was quantified in one portion using Triglyceride E-Test Kits (Wako Pure Chemical Industries Ltd., Osaka, Japan). The cellular proteins were precipitated in the other portion with chilled 6% trichloroacetic acid to remove interfering substances, and then, they were quantified using the Lowry method, with fatty-acid-free bovine serum albumin as the standard. The fat contents were normalized to the protein contents and are expressed as the relative amounts of accumulated lipids in the results.

### 2.4. Quantitative Analysis of Gene Expression

The total RNA (1 μg) extracted from the cells on day 6 of the maturation phase using acid guanidium thiocyanate/phenol/chloroform was reverse transcribed (RT) using M-MLV reverse transcriptase (Ribonuclease H Minus Point Mutant). The single-stranded cDNA was synthesized using oligo-(dT)_15_ and a random 9-mer (Promega Corp., Madison, WI, USA) as primers in the RT reaction. The amount of transcript was determined via qRT-PCR using TB GreenTM Premix Ex TaqTM II (Tli RNaseH Plus) kits (Takara Bio Co., Inc., Kusatsu, Japan) and a Thermal Cycler Dice^TM^ Real Time System (Takara Bio Co., Inc., Kusatsu, Japan) according to the threshold cycle (CT) and ^ΔΔ^CT methods described by the manufacturer. Table 1 shows the oligonucleotides used herein. The cycling program comprised 95 °C for 30 s, 40 cycles at 95 °C for 5 s and 60 °C for 30 s, followed by 95 °C for 15 s and 60 °C for 30 s. Amounts of target gene transcripts were normalized to those of β-Actin. The accession numbers of the target genes are as follows: PPARγ, NM_011146; adiponectin, NM_009605.5; LPL, NM_008509.2; DP1, NM_008962.4; DP2, XM_006526696.5; β-Actin, NM_007393.

### 2.5. Statistical Analyses

All of the results are expressed as means ± standard deviation (SD). The data were statistically analyzed via Student *t*-tests using Excel for Mac (Microsoft Corp., Redmond, WA, USA). The values were considered statistically significant at *p* < 0.05.

## 3. Results

### 3.1. Incubating 3T3-L1 Cells with PGD_2_ or 11d-11m-PGD_2_ during the Differentiation Phase Suppressed MDI-Induced Adipogenesis

**Figure 1:**
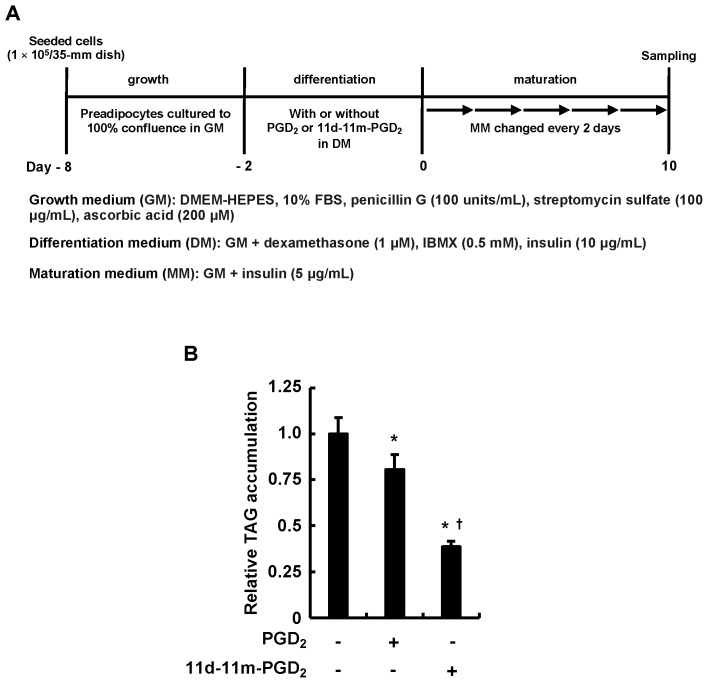
Effects of addition of PGD_2_ or 11d-11m-PGD_2_ during the differentiation phase of 3T3-L1 cells on intracellular TAG accumulation. (**A**) Experimental procedure. We seeded 3T3-L1 cells (1 × 10^5^ per 35 mm dish) containing 2 mL of GM and cultured them until they reached 100% confluence. Confluent cells were incubated for 48 h in 2 mL of DM with or without 1 µM of PGD_2_ or 11d-11m-PGD_2_ each. We replaced DM with 2 mL of fresh MM which was refreshed every 2 days for 10 days. (**B**) Amounts of TAG were analyzed in terminally differentiated mature adipocytes on day 10. Data are expressed as means ± SD of n = 3 experiments. *p* < 0.05 vs. * vehicle (control) and ^†^ PGD_2_ in DM. DM, differentiation medium; GM, growth medium; MM, maturation medium; TAG, triacylglycerol.

We initially examined how the 3T3-L1 cells responded to the addition of PGD_2_ or 11d-11m-PGD_2_ during the differentiation phase, which were pro-adipogenic when added during the maturation phase [36] (Figure 1A). We evaluated the adipogenic differentiation of the 3T3-L1 cells based on the MDI-induced intracellular TAG accumulation. In contrast to our previous findings regarding the pro-adipogenic effects of the addition of PGD_2_ or 11d-11m-PGD_2_ to cells during the maturation phase [36], both PGs attenuated the MDI-induced intracellular TAG accumulation (Figure 1B). Furthermore, 11d-11m-PGD_2_ was more anti-adipogenic than PGD_2_ (Figure 1B). These results suggest that PGD_2_ and 11d-11m-PGD_2_ inhibit the fate-deciding mechanism working in adipogenesis during the differentiation phase.

### 3.2. Downregulated PPARγ Expression Affected the Inhibitory Effects of Addition of PGD_2_ or 11d-11m-PGD_2_ during the Differentiation Phase on MDI-Induced Adipogenesis

**Figure 2:**
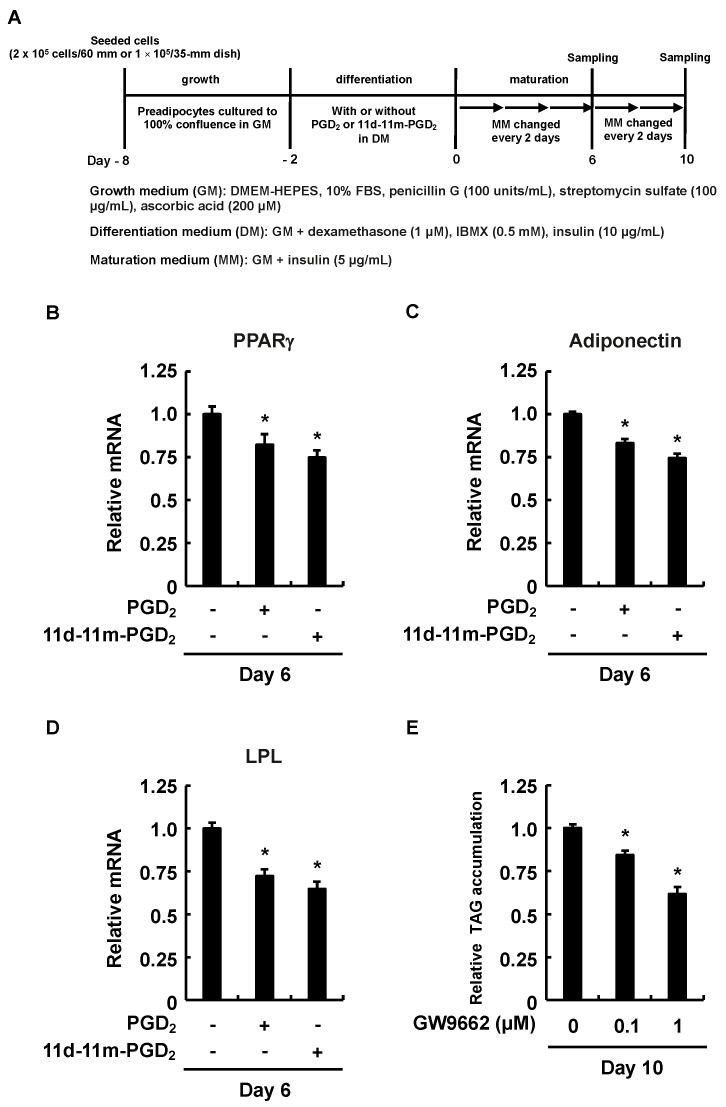
Effects of addition of PGD_2_, 11d-11m-PGD_2_, or PPARγ antagonist, GW9662, during the differentiation phase of 3T3-L1 cells on DP1 and DP2 expression and intracellular TAG accumulation during the maturation phase. (**A**) Experimental procedure. We seeded 3T3-L1 cells (2 × 10^5^ per 60 mm dish) containing 4 mL of GM for quantitative PCR or 3T3-L1 cells (1 × 10^5^ per 35 mm dish) containing 2 mL of GM for analysis of intracellular TAG and incubated them until they reached confluence. Confluent cells were incubated for 48 h during differentiation with 4 mL or 2 mL of DM with or without 1 µM of PGD_2_ or 11d-11m-PGD_2_ and 1 µM of MRE-269. We replaced DM with 4 mL or 2 mL of fresh MM which was refreshed every 2 days. Quantitative PCR results for (**B**) PPARγ, (**C**) adiponectin, and (**D**) LPL on day 6. (**E**) Amounts of TAG were analyzed in terminally differentiated mature adipocytes on day 10. Data are shown as means ± SD of three experiments each for (**B**–**E**). * *p* < 0.05 vs. vehicle (control) in DM. DM, differentiation medium; GM, growth medium; MM, maturation medium; PG, prostaglandin.

We further confirmed whether the reduced level of intracellular TAG accumulation during the maturation phase was reflected in the expression levels of PPARγ, known as one of the master regulators of adipogenesis [39], and the adiponectin and LPL regulated downstream of PPARγ [15,16]. We found that the gene expression levels of PPARγ, adiponectin, and LPL in the 3T3-L1 cells peaked by day six in the maturation phase [40] (Figure 2A). In the maturation phase, the expression level of PPARγ in the 3T3-L1 cells was significantly reduced on day six after the addition of PGD_2_ or 11d-11m-PGD_2_ during the differentiation phase (Figure 2B). The decreased expression of PPARγ also affected the expression levels of its regulated genes, adiponectin and LPL (Figure 2C,D). In particular, LPL is involved in the mechanism by which 3T3-L1 cells take up TAG from the media [19], suggesting that the attenuation of the PPARγ-LPL pathway was reflected in the level of intracellular TAG accumulation. In fact, the addition of the PPARγ antagonist, GW9662, during the differentiation phase of 3T3-L1 inhibited MDI-induced intracellular TAG accumulation during the maturation phase (Figure 2E). Taken together, these results indicate that the addition of PGD_2_ or 11d-11m-PGD_2_ during the differentiation phase suppresses the MDI-induced intracellular TAG accumulation through the downregulation of the PPARγ expression during the maturation phase.

### 3.3. MRE-269 Attenuated the Inhibitory Effects of Addition of PGD_2_ or 11d-11m-PGD_2_ during the Differentiation Phase on MDI-Induced Adipogenesis

**Figure 3:**
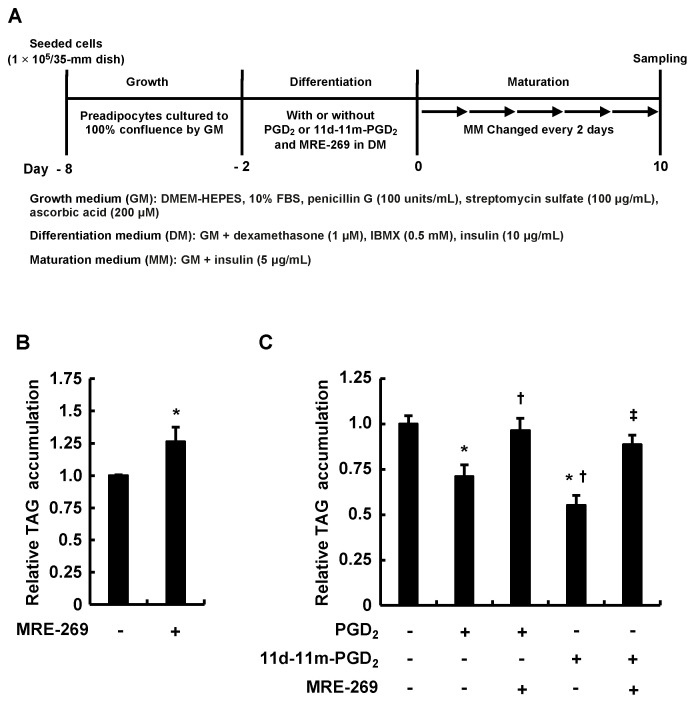
Effects of MRE-269 on preadipocytes cultured with PGD_2_ or 11d-11m-PGD_2_ during the differentiation phase of 3T3-L1 cells. (**A**) Experimental procedure. We seeded and incubated 3T3-L1 cells (1 × 10^5^/dish) in 35 mm dishes containing 2 mL of GM until they became 100% confluent. Confluent cells were incubated for 48 h during differentiation with 2 mL of DM with or without 1 µM of PGD_2_ or 11d-11m-PGD_2_ and 1 µM of MRE-269. We replaced DM with 2 mL of fresh MM which was refreshed every 2 days for 10 days. (**B**,**C**) Amounts of TAG were analyzed in terminally differentiated mature adipocytes on day 10. Data are shown as means ± SD of n = 3 experiments each for (**B**,**C**). *p* < 0.05 vs. * vehicle (control), ^†^ PGD_2_, and ^‡^ 11d-11m-PGD_2_ in DM. DM, differentiation medium; GM, growth medium; MM, maturation medium; PGs, prostaglandins; TAG, triacylglycerol.

To investigate the relationship between the activation of the IP receptor, an origin of the pro-adipogenic signaling of PGI_2_, and the anti-adipogenic effects of PGD_2_ or 11d-11m-PGD_2_, we explored how the coexistence of MRE-269—a highly selective agonist for the IP receptor—with PGD_2_ or 11d-11m-PGD_2_ during the differentiation phase affects the adipogenesis that follows (Figure 3A). In addition to promoting adipogenesis by itself (Figure 3B), in this study, MRE-269 abrogated the anti-adipogenic effects of PGD_2_ or 11d-11m-PGD_2_ (Figure 3C). These findings indicate that the anti-adipogenic effects of PGD_2_ and 11d-11m-PGD_2_ are attenuated depending on the signal from the IP receptor. That is, the levels of PGI_2_ production and IP receptor expression may influence the anti-adipogenic effects of PGD_2_ and 11d-11m-PGD_2_.

### 3.4. DP2 Agonist, but Not DP1 Agonist, Partially Alleviated the Inhibitory Effects of Addition of PGD_2_ or 11d-11m-PGD_2_ during the Differentiation Phase on MDI-Induced Adipogenesis

**Figure 4:**
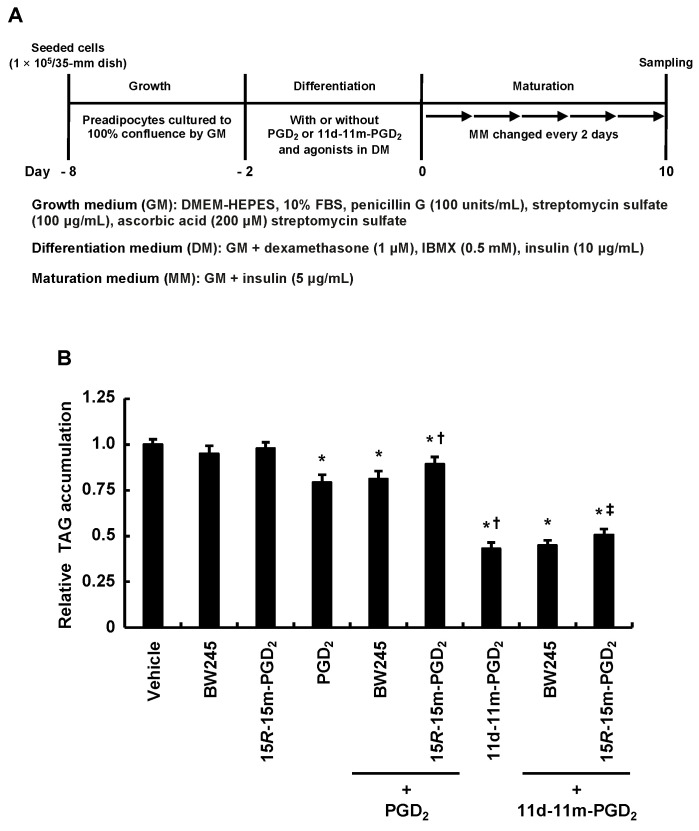
Effects of addition of PGD_2_ or 11d-11m-PGD_2_ and DP1 and DP2 agonists during the differentiation phase of 3T3-L1 cells on intracellular TAG accumulation. (**A**) Experimental procedure. We seeded 3T3-L1 cells (1 × 10^5^ per 35 mm dish) containing 2 mL of GM and cultured them until they reached 100% confluence. Confluent cells were incubated for 48 h during differentiation with 2 mL of DM with or without 1 µM of PGD_2_ or 11d-11m-PGD_2_ and 1 µM of BW245C (DP1 agonist) or 15*R*-15m-PGD_2_ (DP2 agonist). Medium was replaced with 2 mL of fresh MM every 2 days for 10 days. (**B**) Amounts of TAG were analyzed in terminally differentiated mature adipocytes collected on day 10. Data are expressed as means ± SD of n = 3 experiments. *p* < 0.05 vs. * vehicle (control), ^†^ PGD_2_, and ^‡^ 11d-11m-PGD_2_ in DM. DM, differentiation medium; GM, growth medium; MM, maturation medium; PGs, prostaglandins; TAG, triacylglycerol.

We analyzed the inhibitory effects of PGD_2_ and 11d-11m-PGD_2_ on MDI-induced adipogenesis using selective agonists for PGD_2_ receptors, DP1 and DP2, during the differentiation phase, because the activation of these receptors promotes adipogenesis during the maturation phase [35,36] (Figure 4A). The selective DP1 agonist, BW245C, and the selective DP2 agonist, 15*R*-15m-PGD_2_, were used at the previously reported concentrations [35,36]. The selective DP1 agonist, BW245C, did not affect the anti-adipogenic effects of PGD_2_ or 11d-11m-PGD_2_ (Figure 4B). In contrast, the selective DP2 agonist, 15*R*-15m-PGD_2_, slightly but significantly recovered the accumulation of the intracellular TAG that had been decreased by PGD_2_ and 11d-11m-PGD_2_ (Figure 4B). These results suggest that PGD_2_ and 11d-11m-PGD_2_ exert anti-adipogenic effects by inhibiting the DP1 and DP2 activation induced by BW245C and 15*R*-15m-PGD_2_, respectively, during the differentiation phase. That is, PGD_2_ and 11d-11m-PGD_2_ may reduce the sensitivity of DP1 to BW245C and DP2 to 15*R*-15m-PGD_2_.

### 3.5. Incubation of 3T3-L1 Cells with PGD_2_ or 11d-11m-PGD_2_ during the Differentiation Phase Suppressed Expression of DP1 and DP2 during the Maturation Phase

**Figure 5:**
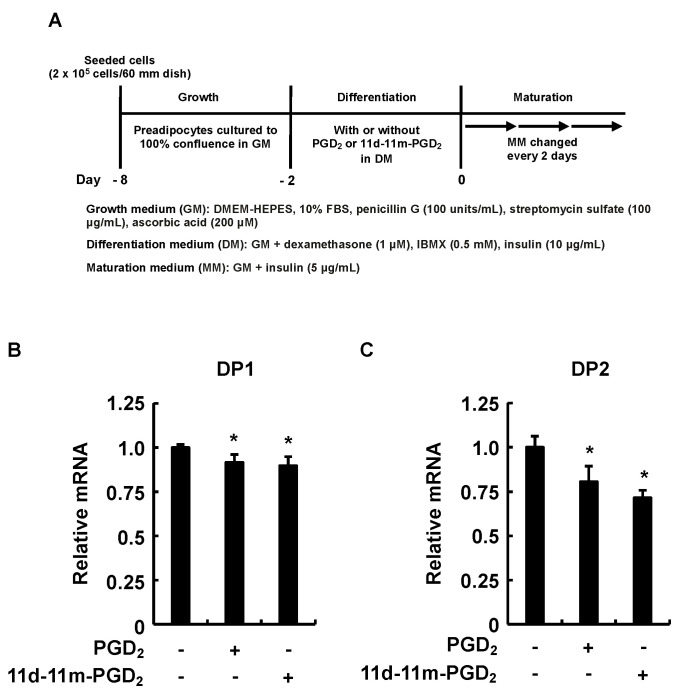
Effects of addition of PGD_2_ or 11d-11m-PGD_2_ during the differentiation phase of 3T3-L1 cells on DP1 and DP2 expression during the maturation phase. (**A**) Experimental procedure. We seeded 3T3-L1 cells (2 × 10^5^ per 60 mm dish) containing 4 mL of GM and incubated them until they reached confluence. Thereafter, cells were incubated in DM with or without 1 µM of PGD_2_ or 11d-11m-PGD_2_ during differentiation and were then cultured for 6 days in MM, which was replaced every 2 days with fresh MM. Quantitative PCR results for (**B**) DP1 and (**C**) DP2 on day 6. Data are shown as means ± SD of three experiments. * *p* < 0.05 vs. vehicle (control) in DM. DM, differentiation medium; GM, growth medium; MM, maturation medium; PG, prostaglandin.

Finally, we evaluated the effects of the addition of PGD_2_ or 11d-11m-PGD_2_ during the differentiation phase on the expression of DP1 and DP2 during the maturation phase (Figure 5A). The incubation of the 3T3-L1 cells with PGD_2_ or 11d-11m-PGD_2_ for two days in the differentiation phase significantly reduced the DP1 and DP2 expression on day six in the maturation phase (Figure 5B,C). As DP1 and DP2 contribute to the promotion of adipogenesis during the maturation phase [36], these results suggest that the addition of PGD_2_ and 11d-11m-PGD_2_ during the differentiation phase exerts anti-adipogenic effects by decreasing the DP1 and DP2 expression during the maturation phase.

## 4. Discussion

**Figure 6:**
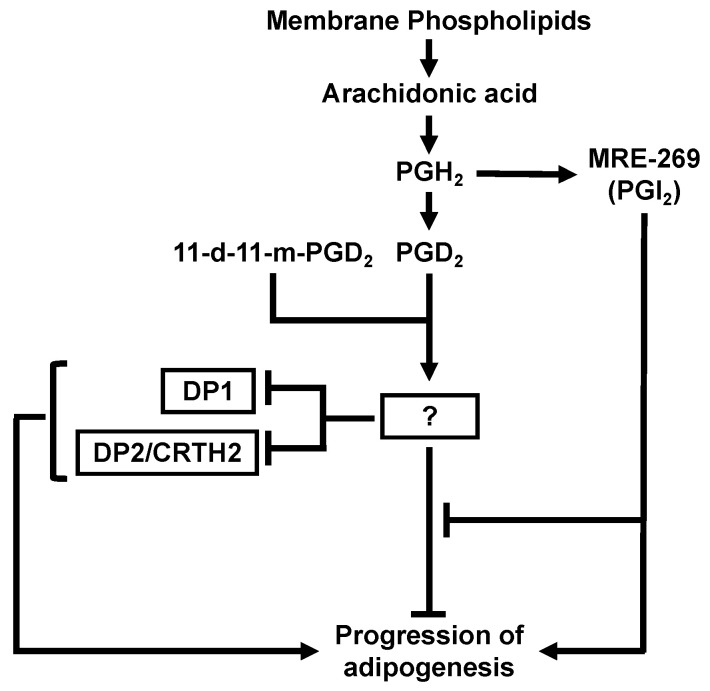
Schematic representation of proposed PGD_2_ or 11d-11m-PGD_2_ function during the differentiation phase and subsequent effects on adipogenesis induced by 3-isobutyl-1-methylxanthine, dexamethasone, and insulin. Interactions between PGD_2_ or 11d-11m-PGD_2_ with unidentified receptor(s) result in the inhibition of adipogenesis by suppressing the expression of DP1 and DP2 or through another pathway independent of DP1 and DP2. 11d-11m-PGD_2_, 11d-11m-prostaglandin D_2_; DP1, D-prostanoid receptor 1; DP2, D-prostanoid receptor 2; PGD_2_, prostaglandin D_2_; PGH_2_, prostaglandin H_2_; Δ^12^-PGJ_2_, Δ^12^-prostaglandin J_2_; PGI_2_, prostaglandin I_2_.

Figure 6 summarizes the present findings. We found that the addition of PGD_2_ and 11d-11m-PGD_2_ during the differentiation phase suppressed adipogenesis during the following maturation phase. These anti-adipogenic effects were disabled by the signal from the IP receptor. In addition, the anti-adipogenic effects of PGD_2_ and 11d-11m-PGD_2_ may be related to the decreased expression of PPARγ during the maturation phase, which may be caused by the reduced sensitivity of the PGD_2_ receptors, DP1 and DP2, during the differentiation phase, as well as by the decreased expression of DP1 and DP2 during the maturation phase. Taken together, PGD_2_ exerts pro-adipogenic effects when its binding to DP1 and DP2 is prioritized during the maturation phase [36], but exerts anti-adipogenic effects, presumably by desensitizing DP1 and DP2, during the differentiation phase. The desensitizing effects may be mediated by the preferential binding of PGD_2_ to unidentified receptor(s), other than DP1 and DP2, which causes the dysfunction of DP1 and DP2 during the differentiation phase, leading to the suppression of adipogenesis in the maturation phase.

The more effective inhibition of adipogenesis via 11d-11m-PGD_2_ in 3T3-L1 preadipocytes may be because PGD_2_ is easily converted to PGJ_2_ derivatives through non-enzymatic dehydration [34], whereas 11d-11m-PGD_2_ is chemically stable with an exocyclic methylene group in place of an 11-keto group. PGJ_2_ derivatives from PGD_2_ may be more pro-adipogenically effective than PGD_2_ during the differentiation phase. In addition, the conversion of PGD_2_ to PGJ_2_ derivatives would reduce the concentration of PGD_2_ acting on the cells. Nevertheless, we found that adipogenesis was inhibited more potently by 11d-11m-PGD_2_ than PGD_2_ because it is more chemically stable and may bind to the target PGD_2_ receptors more strongly than PGD_2_.

We previously showed that DP1 and DP2 are expressed during the differentiation phase [36]. However, in addition to the possibility that PGD_2_ and 11d-11m-PGD_2_ do not act on DP1 or DP2 during the differentiation phase, based on the results of this study, we propose that unidentified receptors for PGD_2_ and 11d-11m-PGD_2_ are involved in the suppression of MDI-induced adipogenesis. Such receptors should have higher affinity for PGD_2_ and 11d-11m-PGD_2_ than DP1 and DP2, or should be more abundantly expressed than DP1 and DP2 during the differentiation phase. This would cause the inhibitory effect of PGD_2_ on adipogenesis to take precedence over its pro-adipogenic effects mediated by DP1 and DP2. In addition, in the present study, the inhibitory effect of PGD_2_ on adipogenesis was stronger, even though ~50% of the initial PGD_2_ would have been transformed to PGJ_2_ derivatives within 6 h at 37 °C in MM [34]. Therefore, PGD_2_ being bound to unidentified receptor(s) would lead to the inhibition of MDI-induced adipogenesis before its conversion into PGJ_2_ derivatives.

Neither PGD_2_ nor 11d-11m-PGD_2_ suppressed the MDI-induced adipogenesis promoted by MRE-269. A positive feedback loop between PPARγ and cytosine–cytosine–adenosine–adenosine–thymidine (CCAAT) box motif/enhancer binding protein alpha (C/EBPα) functions during adipocyte differentiation [41]. MRE-269 activates PPARγ via the IP receptor [33], suggesting that the PPARγ-C/EBPα loop is activated by MRE-269 and PGI_2_. The activation of this loop could alleviate the downregulation of PPARγ caused by PGD_2_ and 11d-11m-PGD_2_ and restore the MDI-induced adipogenesis. Thus, the balance between the signaling from PGD_2_ and PGI_2_ may influence adipogenesis.

According to the findings of the Signaling Pathway Program [42], C/EBPα should bind to the DP1 and DP2 promoters. In addition, C/EBPα forms a regulatory positive feedback loop with PPARγ during adipogenesis [41]. Based on these findings, we predict that PPARγ can regulate DP1 and DP2 expression via C/EBPα. This prediction can be supported by our previous finding that the expression of PPARγ, DP1, and DP2 in the 3T3-L1 cells changed, following a similar trend within ten days of the maturation phase [36]. Furthermore, this finding also suggests that DP1 and DP2 may contribute to adipogenesis during the maturation phase. If DP1 and DP2 also function in a pro-adipogenic manner during the differentiation phase, the addition of PGD_2_ and 11d-11m-PGD_2_ during that period would promote adipogenesis. The present results, however, reveal that the incubation of the 3T3-L1 cells with PGD_2_ or 11d-11m-PGD_2_ for two days in the differentiation phase has anti-adipogenic effects and, furthermore, significantly reduces the expression of PPARγ, DP1, and DP2 on day six in the maturation phase. Thus, if PPARγ activation leads to increased DP1 and DP2 expression during the differentiation phase, then the addition of PGD_2_ and 11d-11m-PGD_2_ during that period would be more pro- than anti-adipogenic. Our present finding that MRE-269 canceled the inhibitory effects of PGD_2_ and 11d-11m-PGD_2_ on adipogenesis during the differentiation phase may also have been due to the increase in DP1 and DP2 expression via the PPARγ activation induced by MRE-269. Taken together, the PPARγ activated by MRE-269 and the IP receptor will increase the expression of C/EBPα, which may promote the upregulation of DP1 and DP2 expression by binding to the DP1 and DP2 promoters. Based on this scheme, we can propose that unidentified PGD_2_ receptor(s) exist at least during the differentiation phase and suppress the expression or transcriptional activity of C/EBPα and/or PPARγ, leading to decreases in the DP1 and DP2 expression levels. However, this requires further investigation, in addition to how PGD_2_ and 11d-11m-PGD_2_ reduce sensitivity to DP1 and DP2 ligands.

Six PG receptors—DP1, DP2, PPARγ, the IP receptor, the FP receptor, and the EP4 receptor—have been reported to be expressed in the differentiation phase of 3T3-L1 cells [23]. Among these, the FP and EP4 receptors, specific to PGF_2α_ and PGE_2_, respectively, exhibit anti-adipogenic effects [32]. Therefore, it could be possible that PGD_2_ or 11d-11m-PGD_2_ inhibit adipogenesis via signaling from the FP and/or EP4 receptors if the compounds can act on these receptors. The FP receptor reduces the transcriptional activity of PPARγ via its phosphorylation through the activation of mitogen-activated protein kinase (MAPK) [43]. The EP4 receptor produces PGF_2α_ by increasing its COX-2 expression [44]. That is, the activation of the EP4 receptor may lead to a decrease in the transcriptional activity of PPARγ, as well as the FP receptor. As PPARγ has an auto-loop mechanism with C/EBPα [41], decreased PPARγ transcriptional activity simultaneously leads to the suppression of its own expression via decreased C/EBPα expression [41]. As a result, the activation of the FP and EP4 receptors would lead to a decrease in the expression of PPARγ, which is similar to the results of the present study. However, if PGD_2_ and 11d-11m-PGD_2_ can bind to EP4 and FP receptors, the addition of PGD_2_ and 11d-11m-PGD_2_ during the maturation phase should inhibit MDI-induced adipogenesis. Our previous results show that the addition of PGD_2_ and 11d-11m-PGD_2_ during the maturation phase promotes MDI-induced adipogenesis [36]. This means that PGD_2_ and 11d-11m-PGD_2_ may not bind to EP4 and/or FP receptors. As described above, it is likely that PGD_2_ and 11d-11m-PGD_2_ do not bind to the previously reported PG receptors expressed in 3T3-L1 cells and are more likely to be a ligand for other receptors.

We focused on insulin signaling as a mechanism by which PGD_2_ and 11d-11m-PGD_2_ inhibit MDI-induced adipogenesis via receptors other than the PG receptors previously reported. Insulin signaling is essential for adipogenesis [45], and insulin was also added during the differentiation phase in the present study. Insulin leads the insulin receptor and its substrate, insulin receptor substrate-1 (IRS-1), to phosphorylate tyrosine residues [46], through which it signals phosphatidylinositol-3 kinase (PI3K) to Akt, ultimately leading to differentiation from preadipocytes to adipocytes [47]. Thus, if PGD_2_ and 11d-11m-PGD_2_ can be involved in signaling to dephosphorylate phosphorylated tyrosine residues in the insulin receptor and IRS-1, the differentiation from preadipocytes to adipocytes should be inhibited. The leukocyte common antigen-related phosphatase receptor (LAR, also known as protein tyrosine phosphatase-RF (PTP-RF)), a transmembrane receptor-type PTP, is a receptor that dephosphorylates the tyrosine-residue-phosphorylated insulin receptor and IRS-1, and has been reported to inhibit the differentiation from preadipocytes to adipocytes as a PTP [48]. However, unlike transmembrane receptor-type protein tyrosine kinases, transmembrane receptor-type PTPs, including LAR, have reduced PTP activity upon the ligand-mediated dimerization of their receptors [49]. That is, if PGD_2_ and 11d-11m-PGD_2_ are ligands for LAR, PGD_2_ may enhance insulin signaling and thereby promote adipogenesis by suppressing PTP activity through LAR dimerization. Therefore, as PGD_2_ and 11d-11m-PGD_2_ were shown to suppress MDI-induced adipogenesis in the present study, it is unlikely that PGD_2_ and 11d-11m-PGD_2_ are ligands for transmembrane receptor-type PTPs, including LAR.

Continuing to focus on insulin signaling, in addition to the phosphorylation of tyrosine residues, IRS-1 has also been reported to be a phosphorylated serine site. For instance, when human IRS-1 is phosphorylated at serine 312 (serine 307 in mouse IRS-1), its interaction with the insulin receptor is disrupted [47]. In addition, phosphorylation at serine 616 in human IRS-1 (serine 612 in mouse IRS-1) results in the attenuation of its interaction with PI3K. Thus, the phosphorylation of these serine residues in IRS-1 causes insulin resistance. Consistent with these results, if PGD_2_ and 11d-11m-PGD_2_ can bind to the receptors that activate molecules that phosphorylate serine residues of IRS-1, as described above, then the differentiation from preadipocytes to adipocytes should be inhibited by this pathway. However, as we have not yet clarified which intracellular signaling pathways are activated by PGD_2_ and 11d-11m-PGD_2_ in 3T3-L1 cells during the differentiation phase, leading to the downregulation of PPARγ expression and anti-adipogenic effects during the maturation phase, we cannot predict which molecules will function as receptor(s) for PGD_2_ and 11d-11m-PGD_2_ in the present situation. Therefore, in the future, the clarification of the intracellular signaling pathway induced by PGD_2_ and 11d-11m-PGD_2_ and the identification of its receptor are expected, using the decreased expression of PPARγ as an indicator.

## 5. Conclusions

PGD_2_ can promote and inhibit adipogenesis, depending on the affinity or expression of DP1 and DP2 and unidentified receptor(s). Therefore, the unknown receptor(s) for PGD_2_ and the regulatory mechanisms of DP1 and DP2 expression should be identified to understand the effects of PGs on the adipogenic program that drives the differentiation of white adipocytes. In addition, in the investigation of the actions of PGD_2_, 11d-11m-PGD_2_ would be a more practical choice.

## Figures and Tables

**Table 1:** Forward (F) and reverse (R) primers for target genes.

GenBank Accession No.	Target Genes	Primers (5→3′)	Length (bp)	Tm (°C)	Product Length (bp)
NM_011146.4	PPARγ	F: CTTCGCTGATGCACTGCCTAT	21	60.81	216
		R: GGGTCAGCTCTTGTGAATGGA	21	60.00	
NM_009605.5	Adiponectin	F: AGCCGCTTATGTGTATCGCT	20	59.61	154
		R: GAGTCCCGGAATGTTGCAGT	20	60.32	
NM_008509.2	LPL	F: TTGCAGAGAGAGGACTCGGA	20	59.96	125
		R: GGAGTTGCACCTGTATGCCT	20	60.04	
NM_008962.4	DP1	F: GAGTCCTATCGCTGTCAGA	19	52.63	100
		R: CCAGAAGATTGCCCAGAAG	19	52.63	
XM_006526696.5	DP2	F: GCGCTATCCGACTTGTTAG	19	55.94	100
		R: GTAGCTTGCAGAAGGTAGTG	20	55.88	
NM_007393.5	β-Actin	F: GCGGGCGACGATGCT	15	59.84	197
		R: TGCCAGATCTTCTCCATGTCG	21	59.86	

## Data Availability

The data presented in this study are available on request from the corresponding author.

## References

[1] Seidell J.C., Halberstadt J. (2015). The global burden of obesity and the challenges of prevention. Ann. Nutr. Metab..

[2] Visscher T.L., Seidell J.C. (2001). The public health impact of obesity. Annu. Rev. Public. Health.

[3] Green H., Kehinde O. (1974). Sublines of mouse 3T3 cells that accumulate lipid. Cell.

[4] Green H., Kehinde O. (1975). An established preadipose cell line and its differentiation in culture. II. Factors affecting the adipose conversion. Cell.

[5] Rangwala S.M., Lazar M.A. (2000). Transcriptional control of adipogenesis. Annu. Rev. Nutr..

[6] Rosen E.D., Spiegelman B.M. (2000). Molecular regulation of adipogenesis. Annu. Rev. Cell Dev. Biol..

[7] Zhu Y., Qi C., Korenberg J.R., Chen X.N., Noya D., Rao M.S., Reddy J.K. (1995). Structural organization of mouse peroxisome proliferator-activated receptor γ (mPPARγ) gene: Alternative promoter use and different splicing yield two mPPARγ isoforms. Proc. Natl. Acad. Sci. USA.

[8] Fajas L., Auboeuf D., Raspé E., Schoonjans K., Lefebvre A.M., Saladin R., Najib J., Laville M., Fruchart J.C., Deeb S. (1997). The organization, promoter analysis, and expression of the human PPARγ gene. J. Biol. Chem..

[9] Tontonoz P., Hu E., Spiegelman B.M. (1994). Stimulation of adipogenesis in fibroblasts by PPARγ2, a lipid-activated transcription factor. Cell.

[10] Wright H.M., Clish C.B., Mikami T., Hauser S., Yanagi K., Hiramatsu R., Serhan C.N., Spiegelman B.M. (2000). A synthetic antagonist for the peroxisome proliferator-activated receptor γ inhibits adipocyte differentiation. J. Biol. Chem..

[11] Camp H.S., Chaudhry A., Leff T. (2001). A novel potent antagonist of peroxisome proliferator-activated receptor γ blocks adipocyte differentiation but does not revert the phenotype of terminally differentiated adipocytes. Endocrinology.

[12] Gurnell M., Wentworth J.M., Agostini M., Adams M., Collingwood T.N., Provenzano C., Browne P.O., Rajanayagam O., Burris T.P., Schwabe J.W. (2000). A dominant-negative peroxisome proliferator-activated receptor γ (PPARγ) mutant is a constitutive repressor and inhibits PPARγ-mediated adipogenesis. J. Biol. Chem..

[13] Barak Y., Nelson M.C., Ong E.S., Jones Y.Z., Ruiz-Lozano P., Chien K.R., Koder A., Evans R.M. (1999). PPARγ is required for placental, cardiac, and adipose tissue development. Mol. Cell.

[14] Rosen E.D., Sarraf P., Troy A.E., Bradwin G., Moore K., Milstone D.S., Spiegelman B.M., Mortensen R.M. (1999). PPARγ is required for the differentiation of adipose tissue in vivo and in vitro. Mol. Cell.

[15] Iwaki M., Matsuda M., Maeda N., Funahashi T., Matsuzawa Y., Makishima M., Shimomura I. (2003). Induction of adiponectin, a fat-derived antidiabetic and antiatherogenic factor, by nuclear receptors. Diabetes.

[16] Schoonjans K., Peinado-Onsurbe J., Lefebvre A.M., Heyman R.A., Briggs M., Deeb S., Staels B., Auwerx J. (1996). PPARα and PPARγ activators direct a distinct tissue-specific transcriptional response via a PPRE in the lipoprotein lipase gene. EMBO J..

[17] Kondo H., Shimomura I., Matsukawa Y., Kumada M., Takahashi M., Matsuda M., Ouchi N., Kihara S., Kawamoto T., Sumitsuji S. (2002). Association of adiponectin mutation with type 2 diabetes: A candidate gene for the insulin resistance syndrome. Diabetes.

[18] Auwerx J., Leroy P., Schoonjans K. (1992). Lipoprotein lipase: Recent contributions from molecular biology. Crit. Rev. Clin. Lab. Sci..

[19] Wise L.S., Green H. (1978). Studies of lipoprotein lipase during the adipose conversion of 3T3 cells. Cell.

[20] Vane J.R., Bakhle Y.S., Botting R.M. (1998). Cyclooxygenases 1 and 2. Annu. Rev. Pharmacol. Toxicol..

[21] Smith W.L., DeWitt D.L., Garavito R.M. (2000). Cyclooxygenases: Structural, cellular, and molecular biology. Annu. Rev. Biochem..

[22] Sugimoto Y., Tsuboi H., Okuno Y., Tamba S., Tsuchiya S., Tsujimoto G., Ichikawa A. (2004). Microarray evaluation of EP4 recep-tor-mediated prostaglandin E2 suppression of 3T3-L1 adipocyte differentiation. Biochem. Biophys. Res. Commun..

[23] Tsuboi H., Sugimoto Y., Kainoh T., Ichikawa A. (2004). Prostanoid EP4 receptor is involved in suppression of 3T3-L1 adipocyte differentiation. Biochem. Biophys. Res. Commun..

[24] Inazumi T., Shirata N., Morimoto K., Takano H., Segi-Nishida E., Sugimoto Y. (2011). Prostaglandin E2-EP4 signaling suppresses adipocyte differentiation in mouse embryonic fibroblasts via an autocrine mechanism. J. Lipid Res..

[25] Miller C.W., Casimir D.A., Ntambi J.M. (1996). The mechanism of inhibition of 3T3-L1 preadipocyte differentiation by prosta-glandin F2α. Endocrinology.

[26] Fujimori K., Ueno T., Nagata N., Kashiwagi K., Aritake K., Amano F., Urade Y. (2010). Suppression of adipocyte differentiation by aldo-keto reductase 1B3 acting as prostaglandin F2α synthase. J. Biol. Chem..

[27] Forman B.M., Tontonoz P., Chen J., Brun R.P., Spiegelman B.M., Evans R.M. (1995). 15-Deoxy-Δ12,14-prostaglandin J2 is a ligand for the adipocyte determination factor PPARγ. Cell.

[28] Kliewer S.A., Lenhard J.M., Willson T.M., Patel I., Morris D.C., Lehmann J.M. (1995). A prostaglandin J2 metabolite binds peroxisome proliferator-activated receptor γ and promotes adipocyte differentiation. Cell.

[29] Mazid M.A., Chowdhury A.A., Nagao K., Nishimura K., Jisaka M., Nagaya T., Yokota K. (2006). Endogenous 15-deoxy-Δ12,14-prostaglandin J2 synthesized by adipocytes during maturation phase contributes to upregulation of fat storage. FEBS Lett..

[30] Hossain M.S., Chowdhury A.A., Rahman M.S., Nishimura K., Jisaka M., Nagaya T., Shono F., Yokota K. (2011). Development of enzyme-linked immunosorbent assay for Δ12-prostaglandin J2 and its application to the measurement of endogenous product generated by cultured adipocytes during the maturation phase. Prostaglandins Other Lipid Mediat..

[31] Massiera F., Saint-Marc P., Seydoux J., Murata T., Kobayashi T., Narumiya S., Guesnet P., Amri E.Z., Negrel R., Ailhaud G. (2003). Arachidonic acid and prostacyclin signaling promote adipose tissue development: A human health concern?. J. Lipid Res..

[32] Rahman M.S. (2019). Prostacyclin: A major prostaglandin in the regulation of adipose tissue development. J. Cell Physiol..

[33] Falcetti E., Flavell D.M., Staels B., Tinker A., Haworth S.G., Clapp L.H. (2007). IP receptor-dependent activation of PPARγ by stable prostacyclin analogues. Biochem. Biophys. Res. Commun..

[34] Mazid M.A., Nishimura K., Nagao K., Jisaka M., Nagaya T., Yokota K. (2007). Development of enzyme-linked immunosorbent assay for prostaglandin D2 using the stable isosteric analogue as a hapten mimic and its application. Prostaglandins Other Lipid Mediat..

[35] Wakai E., Aritake K., Urade Y., Fujimori K. (2017). Prostaglandin D_2_ enhances lipid accumulation through suppression of lipolysis via DP2 (CRTH2) receptors in adipocytes. Biochem. Biophys. Res. Commun..

[36] Rahman M.S., Syeda P.K., Nartey M.N.N., Chowdhury M.M.I., Shimizu H., Nishimura K., Jisaka M., Shono F., Yokota (2018). Comparison of pro-adipogenic effects between prostaglandin (PG)D_2_ and its stable, isosteric analogue, 11-deoxy-11-methylene-PGD_2_, during the maturation phase of cultured adipocytes. Prostaglandins Other Lipid Mediat..

[37] Chowdhury A.A., Hossain M.S., Rahman M.S., Nishimura K., Jisaka M., Nagaya T., Shono F., Yokota K. (2011). Sustained expression of lipocalin-type prostaglandin D synthase in the antisense direction positively regulates adipogenesis in cloned cultured preadipocytes. Biochem. Biophys. Res. Commun..

[38] Hossain M.S., Chowdhury A.A., Rahman M.S., Nishimura K., Jisaka M., Nagaya T., Shono F., Yokota K. (2012). Stable expression of lipocalin-type prostaglandin D synthase in cultured preadipocytes impairs adipogenesis program independently of endogenous prostanoids. Exp. Cell Res..

[39] Lowell B.B. (1999). PPARγ: An essential regulator of adipogenesis and modulator of fat cell function. Cell.

[40] Khan F., Syeda P.K., Nartey M.N.N., Rahman M.S., Islam M.S., Nishimura K., Jisaka M., Shono F., Yokota K. (2016). Pretreatment of cultured preadipocytes with arachidonic acid during the differentiation phase without a cAMP-elevating agent enhances fat storage after the maturation phase. Prostaglandins Other Lipid Mediat..

[41] Wu Z., Rosen E.D., Brun R., Hauser S., Adelmant G., Troy A.E., McKeon C., Darlington G.J., Spiegelman B.M. (1999). Cross-regulation of C/EBPα and PPARγ controls the transcriptional pathway of adipogenesis and insulin sensitivity. Mol. Cell.

[42] Ochsner S.A., Abraham D., Martin K., Ding W., McOwiti A., Kankanamge W., Wang Z., Andreano K., Hamilton R.A., Chen Y. (2019). The Signaling Pathways Project, an integrated ’omics knowledgebase for mammalian cellular signaling pathways. Sci. Data.

[43] Reginato M.J., Krakow S.L., Bailey S.T., Lazar M.A. (1998). Prostaglandins promote and block adipogenesis through opposing effects on peroxisome proliferator-activated receptor γ. J. Biol. Chem..

[44] Fujimori K., Yano M., Ueno T. (2012). Synergistic suppression of early phase of adipogenesis by microsomal PGE synthase-1 (PTGES1)-produced PGE_2_ and aldo-keto reductase 1B3-produced PGF_2α_. PLoS ONE.

[45] Smith P.J., Wise L.S., Berkowitz R., Wan C., Rubin C.S. (1988). Insulin-like growth factor-I is an essential regulator of the differentiation of 3T3-L1 adipocytes. J. Biol. Chem..

[46] White M.F., Kahn C.R. (1994). The insulin signaling system. J. Biol. Chem..

[47] Schmitz-Peiffer C., Whitehead J.P. (2003). IRS-1 regulation in health and disease. IUBMB Life.

[48] Kim W.K., Jung H., Kim D.H., Kim E.Y., Chung J.W., Cho Y.S., Park S.G., Park B.C., Ko Y., Bae K.H. (2009). Regulation of adipogenic differentiation by LAR tyrosine phosphatase in human mesenchymal stem cells and 3T3-L1 preadipocytes. J. Cell Sci..

[49] Welsh C.L., Pandey P., Ahuja L.G. (2021). Protein Tyrosine Phosphatases: A new paradigm in an old signaling system?. Adv. Cancer Res..

